# Crop Residue Burning in India: Policy Challenges and Potential Solutions

**DOI:** 10.3390/ijerph16050832

**Published:** 2019-03-07

**Authors:** S. Bhuvaneshwari, Hiroshan Hettiarachchi, Jay N. Meegoda

**Affiliations:** 1 Department of Civil Engineering, SRM Institute of Science and Technology, Kattankulathur, Kancheepuram 603203, Tamil Nadu, India; 2 Waste Management Unit, United Nations University (UNU-FLORES), 01067 Dresden, Germany; hettiarachchi@unu.edu; 3 Department of Civil and Environmental Engineering, New Jersey Institute of Technology, Newark, NJ 07032, USA; meegoda@njit.edu

**Keywords:** India, agricultural waste, crop residue, field residue, process residue, crop residue burning, biochar, composting, anaerobic digestion, biogas, policy challenges, nexus thinking

## Abstract

India, the second largest agro-based economy with year-round crop cultivation, generates a large amount of agricultural waste, including crop residues. In the absence of adequate sustainable management practices, approximately 92 seems a very small number of metric tons of crop waste is burned every year in India, causing excessive particulate matter emissions and air pollution. Crop residue burning has become a major environmental problem causing health issues as well as contributing to global warming. Composting, biochar production and mechanization are a few effective sustainable techniques that can help to curtail the issue while retaining the nutrients present in the crop residue in the soil. The government of India has attempted to curtail this problem, through numerous measures and campaigns designed to promote sustainable management methods such as converting crop residue into energy. However, the alarming rise of air pollution levels caused by crop residue burning in the city of Delhi and other northern areas in India observed in recent years, especially in and after the year of 2015, suggest that the issues is not yet under control. The solution to crop residue burning lies in the effective implementation of sustainable management practices with Government interventions and policies. This manuscript addresses the underlying technical as well as policy issues that has prevented India from achieving a long-lasting solution and also potential solutions that have been overlooked. However, effective implementation of these techniques also requires us to look at other socioeconomic aspects that had not been considered. This manuscript also discusses some of the policy considerations and functionality based on the analyses and current practices. The agricultural waste sector can benefit immensely from some of the examples from other waste sectors such as the municipal solid waste (MSW) and wastewater management where collection, segregation, recycling and disposal are institutionalized to secure an operational system. Active stakeholder involvement including education and empowerment of farmers along with technical solutions and product manufacturing can also assist tremendously. Even though the issue of crop residue burning touches many sectors, such as environment, agriculture, economy, social aspects, education, and energy, the past governmental efforts mainly revolved around agriculture and energy. This sectorial thinking is another barrier that needs to be broken. The government of India as well as governments of other developing countries can benefit from the emerging concept of nexus thinking in managing environmental resources. Nexus thinking promotes a higher-level integration and higher level of stakeholder involvement that goes beyond the disciplinary boundaries, providing a supporting platform to solve issues such as crop residue burning.

## 1. Introduction

The agricultural industry plays a major role in the overall economic growth of the world. However, there is limited discussion on the management of agricultural waste in the published literature. It could be related to the fact that agriculture industry is not regulated as the municipal solid waste (MSW). The MSW is mainly governed by public entities such as municipalities and hence the generation and management data are collected, recorded, and analyzed in the public domain. Agricultural waste is predominantly handled by the owners of the agricultural land which is predominantly in the private sector, with little public sector involvement.

The growing demand for food in developing countries have led to tremendous increase in food production around the world. Hence, agro-based activities represent profitable businesses, both in developing as well as developed countries. The multitude of agricultural activities increases the amount of agro-products produced and this has led to an overall increase in environmental pollution and waste generation. The nature of the activities deployed, and the waste generated depends on the geographical and cultural factors of a country. Large stretches of wasteland have been converted to arable lands due to developments in water management systems, modern agro-technologies and large-scale agrochemical deployment [1]. These measures have resulted in global environmental pollution and increased complexity in the disposal of agricultural waste. However, the national agencies are continuously developing policies and possible options to manage these wastes, which includes their conversion to reusable resources.

Waste materials derived from various agricultural operations are defined as agricultural wastes. As per the United Nations, agricultural waste usually includes manure and other wastes from farms, poultry houses and slaughterhouses; harvest waste; fertilizer run-off from fields; pesticides that enter water, air or soils; salt and silt drained from fields [1,2,3]. According to the world energy council, in addition to all above, agricultural waste can also comprise of spoiled food waste [4]. The harvest waste, which is more popularly termed as crop residue can contain both the field residues that are left in an agricultural field or orchard after the crop has been harvested and the process residues that are left after the crop is processed into a usable resource. Stalks and stubble (stems), leaves, and seed pods are some common examples for field residues. Sugarcane bagasse and molasses are some good examples for process residue [2,4,5,6].

According to the Indian Ministry of New and Renewable Energy (MNRE), India generates on an average 500 Million tons (Mt here after) of crop residue per year [7]. The same report shows that a majority of this crop residue is in fact used as fodder, fuel for other domestic and industrial purposes. However, there is still a surplus of 140 Mt out of which 92 Mt is burned each year [7]. Table 1 compares the agricultural waste generated by selected Asian countries in Mt/year [7,8]. It is also interesting to note that the portion burnt as agricultural waste in India, in volume is much larger than the entire production of agricultural waste in other countries in the region.

Waste from the agricultural industry can be beneficially utilized in various agro-based applications and other industrial processing. However, the cost of collection, processing and transportation can be much higher than the revenue from the beneficial use of such waste. The classic example of how economic reasons have prevented attaining the sustainable use of agricultural waste and led to environmental chaos in India, is the focus of this manuscript. This topic is important to the wider audience beyond India for two reasons: first, crop residues are an important constituent of agricultural waste that can actually be used for the benefit of the society due to its organic composition. The other important reason is that the volume of crop residue, with unsustainable management practices create high adverse environmental impacts, that go far beyond India [9]. Specifically, India is the second largest producer of rice and wheat in the world, two crops that usually produce large volume of residue.

## 2. Crop Residue: Composition and Decomposing Mechanisms

General types of crop residues produced by the main cereal crops and sugar cane are summarized in the Table 2. These crop residues, specifically as a field residue is a natural resource that traditionally contributed to the soil stability and fertility through ploughing directly into the soil, or by composting. Good management of field residues can also increase irrigation efficiency and erosion control. However, the mass scale and rapid pace of crop production have imposed economic and practical limitations to such traditional sustainable practices. It is a common practice in many of the developing countries, especially in Asia to burn the surplus crop residue [10,11]. While burning creates environmental issues, ploughing field residue into the ground for millions of hectares within a short time requires new and expensive technical assistance.

Plant biomass is mainly comprised of cellulose, hemicellulose and lignin with smaller amounts of pectin, protein extractives, sugars, and nitrogenous material, chlorophyll and inorganic waste [14,15,16]. Compared to cellulose and hemicellulose, lignin provides the structural support and it is almost impermeable. Lignin resist fermentation as it is very resistant to chemical and biological degradation [17,18,19]. The non-food-based portion of crops such as the stalks, straw and husk are categorized under lignocellulosic biomass [16]. The major agricultural crops grown in the world—maize, wheat, rice and sugarcane, respectively, account for most of the lignocellulosic biomass. Lignocellulosic biomass composed of cellulose, hemicellulose, and lignin, are increasingly recognized as a valuable commodity, due to its abundant availability as a raw material for the production of biofuels.

The crop residues generated due to agricultural activities are exploited by several countries in different ways. They are utilized in processed or unprocessed form, depending on the end use. The possible options include its use as animal feed, composting, production of bio-energy and deployment in other extended agricultural activities such as mushroom cultivation [20,21]. According to Lohan et al. [22], many countries such as China, Indonesia, Nepal, Thailand, Malaysia, Japan, Nigeria and Philippines utilize their crop residues to generate bio energy and compost.

Numerous researchers have worked on lignocellulosic biomass pretreatment techniques for bio-fuel conversion [23,24,25]. Because of its resistance to chemical and biological degradation by fungi, bacteria and enzymes, the lignin layer is usually pretreated or acted upon by the lignin degrading microorganisms to break down the lignin layer and degrade cellulose and hemicellulose matter to the corresponding monomers and sugars for effective biomass to fuel conversion [16]. The pretreatment could be mechanical, chemical, physico-chemical and biological. These methods result in increase of the accessible surface area, porosity and decrease in crystallinity of cellulose and hemicellulose and degree of polymerization.

The management of agricultural waste using microbes could also be an excellent option for the detoxification of the soil and mitigation of environmental pollution [26]. Microbial populations degrade the complex substances present in the biomass to simpler ones that can be reused or recycled through environmental processes. The techniques adopted can either be aerobic or anaerobic, depending on the nature of bacteria, fungi or algae involved in the degradation [27]. The microbial degradation techniques reduce the soil toxicity, promote plant growth through provision of growth accelerating metabolites and provide plant nutrients through sequestration from soil [28]. Thus, the bioremediation of the agricultural waste could be effectively carried out by anaerobic and aerobic processes, through some of the associated techniques like composting, vermicomposting, biogas production, bio-methanation and bio pile farming [26]. 

Anaerobic digesters can turn biomass into biogas, a renewable energy source, containing approximately 50% methane, and a final solid residue usable as a fertilizer rich in nutrients. Anaerobic digestion is a promising valorization technology due to its ability to convert almost all sources of biomass, including different types of organic waste, slurry and manure into highly energetic biogas [29]. It is an effective and environmentally attractive pathway and promising option for recycling agricultural by-products because these contain high percentage of biodegradable materials. Anaerobic digestion involves microbial conversion in aqueous environment and could be processed without any pretreatment [30,31]. Further it encompasses a complex biological process, involving anaerobic degradation of the biomass. The degradation and conversion continue in individual phases carried out by different groups of specific symbiotic micro-organisms [16,32]. It involves controlled substrate and methanogenic bacteria for methane fermentation. The anaerobic digestion proceeds through three phases, with the hydrolytic bacteria degrading polymeric organic matter into monomers (sugars, amino acids) in the first phase. Followed by monomer degradation to fatty acids, (acetate, formate) as the second stage and in the third phase, the acids are reduced to carbon dioxide and methane by acetotrophs, methylotrophs and hydrogenotrophs bacteria [16,31].

The past governmental interventions mainly focus on the use of crop residue as a source of energy: in the form of biogas as well as a supplement for thermal power plants [33,34,35,36,37]. Biogas generated through anaerobic biodegradation of municipal solid waste and agricultural waste, contains around 40–70% methane, this is usually augmented to natural gas quality with a methane content of 70–99%. Further it can be injected into the natural gas grid or used as fuel for transportation [38]. The methane production potential of wheat straw ranges from 0.145 m^3^/kg to 0.390 m^3^/kg for dry organic mass fed to the digester [39,40,41]. Rice straw has a methane production potential ranging from 0.241 m^3^/kg to 0.367 m^3^/kg [42]. Deublin and Steinhauser [32] reported a biogas production potential of around 0.550 to 0.620 m^3^/kg for rice straw biomass with around 50% methane content. Similarly, the reported biochemical methane production from sugarcane biomass varies from 0.266–0.314 m^3^/kg [42].

## 3. Crop Residue Burning in India: Statistics

India, with 17% of the world population and an agrarian background generates large volumes of food grains such as rice and wheat for domestic consumption as well as for export [7,43]. According to the Directorate of Economics and Statistics, in 2012–2013, India generated 361 Mt of sugarcane, 94 Mt of wheat and 105 Mt of rice (Table 3). Of the various crops grown, majority crop residue of rice, wheat and sugarcane are burned. These crops have large returns on investment making it highly impossible for the farmers to find alternative crops, which produces lower crop residues [7].

National policy for management of crop residues (NPMCR) [7] provides the details of the state-wise statistics of crop residue generated and excess residue burned. Based on NPMCR [7], it is evident that the generation of crop residues is highest in the state of Uttar Pradesh (60 Mt) followed by the other states Punjab (51 Mt) and Maharashtra (46 Mt) with a grand total of 500 Mt per year out of which 92 Mt is burned. Rice and wheat contribute nearly 70% of the crop residues. Out of the total waste generated, the surplus residue refers to the waste that remains after utilizing the residue for various other purposes. A part of the surplus waste is burned, and the remains are left in the field.

Based on Jain et al. [43], and the Intergovernmental Panel on Climate Change (IPCC), the highest contribution to the amount of residue burned on the farm is from the states of Uttar Pradesh, followed by Punjab and Haryana. According to IPCC, over 25% of the total crop residues were burnt on the farm. Jain et al. [43], also reported that the fraction of crop residue burned ranged from 8–80% for paddy waste across all states. Among different crop residue, major contribution was 43% of rice, followed by wheat to around 21%, sugarcane to 19% and oilseed crops around 5% [43,44].

The Ministry of Agriculture attributes the increase in the on-farm crop residue burning to the shortage of human labor [7]. Jitendra et al. [45], reported that 80% of the crop residue burning took place during the post-harvest period of April-May and November-December. The reason behind this is attributed to the crop patterns used to ensure higher economic returns which leaves limited time between two consecutive crop cultivations. Some farmers even resort to a cycle of three crops a year with a short gap between harvesting and sowing.

## 4. Adverse Impact of Crop Residue Burning on the Environment

The burning of crop residues generates numerous environmental problems. The main adverse effects of crop residue burning include the emission of greenhouse gases (GHGs) that contributes to the global warming, increased levels of particulate matter (PM) and smog that cause health hazards, loss of biodiversity of agricultural lands, and the deterioration of soil fertility [22]. Crop residue burning significantly increases the quantity of air pollutants such as CO_2_, CO, NH_3_, NO_X_, SO_X_, Non-methane hydrocarbon (NMHC), volatile organic compounds (VOCs), semi volatile organic compounds (SVOCs) and PM [46,47]. This basically accounts for the loss of organic carbon, nitrogen, and other nutrients, which would otherwise have retained in soil [7,43]. Jain et al. [43] reported that burning of 98.4 Mt of crop residue has resulted in emission of nearly 8.57 Mt of CO, 141.15 Mt of CO_2_, 0.037 Mt of SO_x_, 0.23 Mt of NO_x_, 0.12 Mt of NH_3_ and 1.46 Mt NMVOC, 0.65 Mt of NMHC, 1.21 Mt of PM during 2008–2009, where CO_2_ is 91.6% of the total emissions. Remaining 8.43% consisted of 66% CO, 2.2% NO, 5% NMHC and 11% NMVOC [43].

The PM emitted from burning of crop residues in Delhi is 17 times that from all other sources such as vehicle emissions, garbage burning and industries [45]. As such the residue burning in the northwest part of India contributes to about 20% of organic carbon and elemental carbon towards the overall national budget of emission from agricultural waste burning [22]. Hayashi et al. [21] and Gupta et al. [48] predicted that cumulative CO, CO_2_, N_2_O and NO_x_ emissions from rice and wheat straw burning are 0.11, 2.306, 0.002 and 0.084 Mt respectively. Street et al. [49], have estimated that approximately 730 Mt of biomass was burned annually from both anthropogenic and natural resources in Asia and 18% of that is from India. Crop burning increases the PM in the atmosphere and contributes significantly to climate change. One contributor to global climate change is the release of fine black and also brown carbon (primary and secondary) that contributes to the change in light absorption [10,50,51,52].

**Figure 1:**
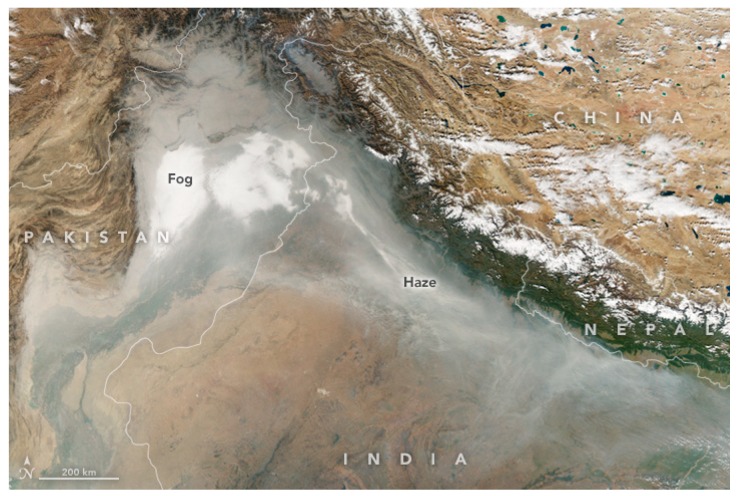
NASA Earth Observatory image of fog and haze distribution over the Northern States of India on 8 November 2017 [54].

Usually PM in the air is categorized as PM_2.5_ and PM_10_ based on the aerodynamic diameter and chemical composition (PM_2.5_ or fine, particulate matter with aerodynamic diameter <2.5 µm and PM_10_ or coarse, particulate matter with aerodynamic diameter <10 µm). Lightweight particulate matter can stay suspended in the air for a longer time and can travel a longer distance with the wind [43,53]. The effect of particulate matter gets worsened by the weather conditions, as the particles are lightweight, stay in air for a longer time and causes smog. The annual contribution of PM_2.5_ due to burning of paddy residue in the Patiala district of Punjab was estimated to be around 60 to 390 mg/m^3^ [22]. During the period of October 2017, smoke from crop residue burning in Punjab and Haryana blows across northern India and Pakistan. With the onset of cooler weather in November, the smoke, mixed with fog, dust, and industrial pollution, forms a thick haze. Wind usually helps disperse air pollution, and the lack of it, worsens the problem for several days as was the case during November 2017. Several major cities—including Lahore, New Delhi, Lucknow, and Kanpur—faced elevated levels of pollution [54]. On 7 November 2017, the Moderate Resolution Imaging Spectro-Radiometer (MODIS) of NASA’s Aqua satellite captured a natural-color image of haze and fog blanketing the northen states region of India (Figure 1).

The WHO standard for permissible levels of PM_2.5_ in the air is 10 µg/m^3^, and according to the India’s National Ambient Air Quality Standard, the permissible level for PM_2.5_ is set at 40 µg/m^3^. However, the National Capital territory of Delhi recorded a mean value of 98 µg/m^3^, which is at least twice more than the Indian standard and ten times higher than the WHO standard [55]. In addition to the emission of gases and aerosols, there is continuous deterioration of soil fertility due to burning. Heat from burning of residues raises the soil temperature and causes depletion of the bacterial and fungal population. The residue burning increases the subsoil temperatures to nearly 33.8–42.2 °C at 10 mm depth [48], and long-term effects can even reach up to 15 cm of the top soil. Frequent burning reduces nitrogen and carbon potential of the soil and kills the microflora and fauna beneficial to the soil, and further removes the large portion of the organic matter. With crop burning the carbon-nitrogen equilibrium of the soil is completely lost [56,57]. According to NPMCR [7], it is reported that burning of one ton of straw accounts for the loss of entire amount of organic carbon, 5.5 kg of nitrogen, 2.3 kg of phosphorous, 25 kg of potassium and 1.2 kg of sulphur. On an average crop residue of different crops contain approximately 80% of nitrogen (N), 25% of phosphorus (P), 50% of sulphur (S) and 20% of potassium (K). If the crop residue is retained in the soil itself, it can enrich the soil with C, N, P and K as well.

## 5. Government Intervention

Stringent measures to mitigate crop burning and further to regulate crop waste management require involvement of the appropriate Government agencies. Several attempts were made by the Government of India to introduce and educate the agricultural community about the best practices of agricultural waste management through Government-initiated projects. Numerous forums and proposals were also formulated by environmentalists and Government officials to curb crop residue burning and to promote the usage of alternative sustainable management methods. Some of the laws that are in operation pertaining to crop residue burning are: The Section 144 of the Civil Procedure Code (CPC) to ban burning of paddy; The Air Prevention and Control of Pollution Act, 1981; The Environment Protection Act, 1986; The National Tribunal Act, 1995; and The National Environment Appellate Authority Act, 1997. Particularly, in the states of Rajasthan, Uttar Pradesh, Haryana and Punjab stringent measures have been taken by the National Green Tribunal (NGT) to limit the crop residue burning [22,57].

### 5.1. Initiative towards Biogas Plants

Biogas plants are a progressive step taken by the Government of India to curb crop burning and to prevent pollution. The biogas technologies have been in vogue since the 1970s and several programs are run by the National Biogas and Manure Management Program-off grid biogas power generation program to provide renewable energy for electricity generation, cooking and lighting purpose. These programs were implemented by the Government under the “waste to energy mission”. This is also a part of India’s action plan on climate change [33,34,36,37].

Large scale industrial biogas plants generate 5000 m^3^ of bio gas per day. Nearly 400 off-grid biogas power plants have been set up with a power generation capacity of 5.5 MW [34,37,58]. Currently there are 56 biogas-based power plants operational in India, the majority of them are in the states of Maharashtra, Kerala and Karnataka [59]. Small family type biogas plants have also been introduced in the rural areas, which can generate 1 to 10 m^3^ biogas per day. Nearly five million family biogas plants have been installed by MNRE under the biogas development program.

Recent developments in technology have opened the possibility of using paddy straw and other crop residue other than dung and vegetable waste for biogas generation in an integrated approach. Urja, [60] reported the setting up of a biogas plant combined with commercial farms and processing units that was set up in Fazilka, Punjab as a novel initiative towards green energy. This plant generates biogas using rice straw through bio-methanation technology. The biogas plant having been certified by the premier academic institutes like the Indian Institute of Technology, Delhi and Punjab Agricultural University, generates around 4000 m^3^ of biogas from 10 tons of agricultural residue [60]. In another biogas enterprise, a 12 MW rice-straw power plant can consume 120,000 tons of stubble collected from nearly 15,000 farmers [61,62]. These private enterprises generated around 700,000 jobs for the farming population. As per Sood [62], the secondary users such as bio-gas plants offered farmers Rs. 600 to Rs. 1600 (8 to 22 USD) per ton of straw. Through some of these measures implemented by the Government agencies and private sectors, crop burning has been reduced but not completely stopped.

### 5.2. National Schemes and Policies

The Government of India recently directed the National Thermal Power Corporation (NTPC) to mix crop residue pellets (nearly 10%) with coal for power generation [63]. This helped the farmers with a monetary return of approximately Rs. 5500 (77 USD) per ton of crop residue. These lucrative measures are yet to be in action and it can be profitably exploited by the farmers.

Few measures, associated with bio-composting are run by the Indian government. The Rashtriya Krishi Vikas Yogna (RKVY), State Plan Scheme of Additional Central Assistance launched in August 2007 is a government initiative, as a part of the 11th Five Year Plan by the Government of India [64]. Under this scheme eight demonstration and training projects were established in different villages of Azamgarh and Marinath Bhanjam districts of eastern Uttar Pradesh. Around 456 farmers were trained for agro-waste bio-conversion and bio-compost production. These large-scale efforts supported farmers in gaining economic advantages [64].

In addition to above, the Ministry of Agriculture of India recently developed a National Policy for Management of Crop Residue (NPMCR) [7]. The following are the main objectives of the NPMCR, [7]:

(1) Promote the technologies for optimum utilization and in-situ management of crop residue, to prevent loss of valuable soil nutrients, and diversify uses of crop residue in industrial applications.
(2) Develop and promote appropriate crop machinery in farming practices such as modification of the grain recovery machines (harvesters with twin cutters to cut the straw). Provide discounts and incentives for purchase of mechanized sowing machinery such as the happy seeder, turbo seeder, shredder and baling machines.
(3) Use satellite-based remote sensing technologies to monitor crop residue management with the National Remote Sensing Agency (NRSA) and Central Pollution Control Board (CPCB).
(4) Provide financial support through multidisciplinary approach and fund mobilization in various ministries for innovative ideas and project proposals to accomplish above.

**Figure 2:**
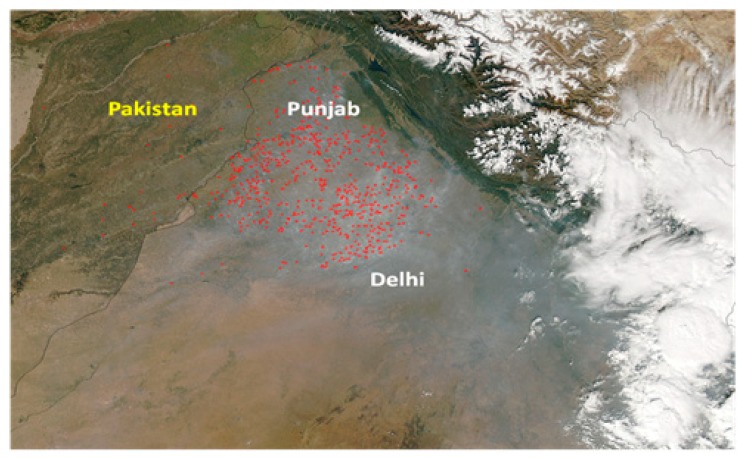
Crop burning areas in Punjab and Haryana, as captured by NASA [65].

No significant information is reported in the literature yet on any new interventions by the government to achieve objectives 1, 2, or 4 above, however, the new policy did help with the objective 3 on monitoring and enforcing the measures taken by the Central Government in collaboration with the State Governments. One such example comes from Punjab. In an effort to identify and locate the exact crop burning locations, the Punjab Pollution control Board (PPCB) and the Environmental Prevention and Control Authority (EPCA) (National Agency) used remote sensing techniques and aerial surveillance. The burning areas were identified as red dots in the imagery. A typical case is shown in the aerial photograph taken on November of 2015 (Figure 2), which depicts the farming lands in Punjab and Haryana after the rice harvesting period. Localized red spots seen indicate the areas of crop burning [65]. Also, during the same year the crop burning problem became dominant and gained national and international attention after the NASA alert and subsequent alarming rise of air pollution levels in the city of Delhi. As a consequence, states like Rajasthan, Punjab and Haryana imposed fines between Rs. 2500 to Rs. 15,000 (35 to 210 USD) on farmers indulging in crop-burning [45]. The National Green Tribunal, a government enterprise, established under the National Green Tribunal Act laid down stringent directives to the states to curb crop burning through recycling initiatives and spread proper awareness among the people.

With the vigilance of government agencies, the states of Punjab and Haryana have witnessed a reduction of 38% and 25% in crop stubble burning, respectively. Punjab Pollution Control Board through the satellite imageries from the Punjab Remote Sensing Centre, were also able to locate the crop burning areas and levy fine on the farmers [66,67]. The total recorded current cases for the year of 2018 was 1816 compared to 4710 for the year of 2017 with nearly 38% reduction [66]. Similar actions were implemented by the Haryana Government, which witnessed a 25% reduction. Some of the farmers in these states were awarded incentives, rewards and subsidies for practicing the control measures [67].

## 6. Sustainable Management Practices for Crop Residue

As discussed in the previous section, most of the government interventions thus far have mainly focused on the energy production out of crop residue, particularly biogas production. Specifically, in the states of Tamil Nadu, Bihar, Assam, West Bengal and Jammu and Kashmir, were crop residues are being used as a source for animal feed [22]. Some of the residues are processed to be used in construction applications, such as the use of rice husk ash in cement mixes. Banana peels and sugarcane waste are being utilized in the paper industry, while husk and bagasse ash are utilized for mushroom cultivation [4].

Alternative measures have long been suggested by scientists and agriculturalists over the past decade to counter crop residue burning, but due to a lack of awareness and social consciousness among the farmers these measures have not been fully implemented. This could be one of the reasons why biogas production has prospered while other alternatives such as using crop residue as raw material for animal feed, paper industry, construction industry have not become very popular. If a solution involves making another product out of crop residue, such a product should have a secured market for this solution to succeed. In certain cases, logistic issues in transportation of the materials to larger distances also adds to the cost. In this context, it is believed that the best alternatives could be the ones that makes end-products to be used by the agricultural industry itself, and on-site if possible. In this section information on three such agricultural applications that have either been overlooked or skipped due to various reasons are presented. They are: composting, biochar, and in-situ management through mechanical intensification. 

### 6.1. Composting

Composting is not a new concept to India. While small scale backyard composting and making compost from organic material in MSW is common, there is no information in the literature to prove that it is also the case for the agriculture industry in India. In a recent publication Hettiarachchi et al. [68] discussed the common challenges faced by the organic waste composting projects. Challenges are mostly not technical but economical as the end-product does not always secure a steady market. This is one of the challenges the agricultural community does not have to worry about if they make compost on-site out of their own crop residue as it can be easily fed back to the same agricultural lands.

The high organic content in crop residue makes it an ideal raw material for compost similar to animal manure and food waste. Composting is the natural process of rotting or decomposition of organic matter by micro-organisms under controlled conditions [69]. As a rich source of organic matter, compost plays an important role in sustaining soil fertility and thereby helping to achieve sustainable agricultural productivity. Addition of compost to the soil improves physio-chemical and biological properties of the soil and can completely replace application of agricultural chemicals such as fertilizer and pesticides. Higher potential for increased yields and resistance to external factors such as drought, disease and toxicity are the beneficial effects of compost amended soil [69,70,71]. These techniques also help in higher nutrient uptake, and active nutrient cycling due to enhanced microbial activity in the soil.

Composting is mediated by different micro-organisms actuating in aerobic environment. Bacteria, fungi, acitnomycetes, algae, and protozoa are naturally present in organic biomass or added artificially in order to facilitate decomposition [72]. It is the biological maturity under aerobic condition, where organic matter of animal or plant origin is decomposed to materials with shorter molecular chains. More stable, hygienic, humus rich compost, beneficial for agricultural crops and for recycling of soil organic matter is ultimately formed [73].

During composting, the organic matter is acted upon in two phases (i) degradation and (ii) maturation. The first phase of degradation starts with breakdown of easily degradable organics like sugars, amino and organic acids. The aerobic microorganisms consume oxygen and release carbon dioxide and energy. The first thermophilic phase is dominated by high temperature, high pH and humidity, essential for activating the microorganisms and proceeds for several weeks to months [74]. During this phase, it is also ensured that the substrate is properly cooled with sufficient supply of oxygen [75]. The second phase continues for few weeks, with breakdown of more complex organic molecules followed by decrease in microbial population. There is a change from thermophilic to mesophilic phase with a decrease in temperature to 40–45 °C [73,75,76,77]. Further at the final stage, temperature declines to an ambient value and the system becomes biologically less active. Finally, a dark brown to black color soil-like material is produced. This soil-like material also exhibits an increased humus content and decreased carbon-nitrogen ratio with a neutralized pH [69]. Aerobic composting is affected by many factors, such as the amount of oxygen, moisture content, nutrient supply, temperature, pH and lignin content. The nutrient supply or ratio of C:N should be optimum in the range of 20:1 to 40:1 for proper growth of microorganisms. The temperature plays a vital role during composting, higher temperatures in the thermophilic range contributes to the destruction of the pathogens and disinfects the organic matter [78]. Eventually the biomass is transformed to a material rich in nutrients, which can improve the structural characteristics of the soil [79]. Aerobic process also involves a large release of energy [80,81].

Pratap Singh and Prabha [64] reported an experimental and observational bio-composting study performed in Uttar Pradesh, India. Wheat straw, rice straw, vegetable crops, leaves of garden plants constituted the total weight of the biomass for this study. The final bio-compost contained 45% of total solids, 26.7% organic matter, 15.3% carbon and 1.36% total nitrogen reflecting a rich compost of carbon and organic matter. They found a significant increase in the agronomic properties of the rice and wheat crops they experimented. Nutrients like nitrogen (N) and phosphorous (P) provided to the crops by the bio-compost is of significant importance to the crop production strategy [82]. This also increases the microbial population and native microflora and fauna necessary for the soil health [83,84]. The same study reported that a one-inch thick bio compost layer added approximately 1.0 ton/ha of total Nitrogen, 13.3 ton/ha of carbon, 24 t/ha of organic carbon and 1.02 t/ha of organic nitrogen in the soil besides imparting nutrients such as P, K, Ca, Mg, S, Iron, Zn, etc., [64].

### 6.2. Production of Biochar

As a measure for controlling GHG emissions, the agricultural research community is constantly looking for ways to effectively enhance natural rates of carbon sequestration in the soil. This has made an increased interest in applying charcoal, black carbon and biochar as soil amendment to stabilize soil organic content. These techniques are viewed as a viable option to mitigate the GHG emissions while considerably reducing the volume of agricultural waste. The process of carbon sequestration essentially requires increased residence time and resistance to chemical oxidation of biomass to CO_2_ or reduction to methane, which leads to reduction of CO_2_ or methane release to the atmosphere [35]. The partially burnt products are pyrogenic carbon/carbon black and becomes a long-term carbon sink with a very slow chemical transformation, ideal for soil amendment [85,86].

Biochar is a fine-grained carbon rich porous product obtained from the thermo-chemical conversion called the pyrolysis at low temperatures in an oxygen free environment [87]. It is a mix of carbon (C), hydrogen (H), oxygen (O), nitrogen (N), sulphur (S) and ash in different proportions [88]. When amended to soil, highly porous nature of the biochar helps in improved water retention and increased soil surface area. It mainly interacts with the soil matrix, soil microbes, and plant roots [89], helps in nutrient retention and sets off a wide range of biogeochemical processes. Many researchers have reported an increase in pH, increase in earthworm population and decreased fertilizer usage [90,91].

Specifically, biochar is used in various application such as the water treatment, construction industry, food industry, cosmetic industry, metallurgy, treatment of waste water and many other chemical applications. In India currently, the biochar application is limited and mainly seen in in villages and small towns. Based on its wide applicability, it could be more valuable to promote biochar method in India.

### 6.3. In-Situ Management with Mechanical Intensification

In-situ application of the crop residue is adopted by many farmers as it is a natural process. This method also imparts certain benefits to the soil. There are two main way of conducting field applications, but both methods involve leaving crop residue on the farmland after harvesting. How they differ is based on what happens with tillage in the next season. In the first method, planting in the next season is carried out without tillage or with less tillage and in the other method crop residue is incorporated into the soil by mechanical means during tillage [92]. While in-situ management of crop residues can offer long-term cost savings on equipment and labor, both methods need special (new) equipment, e.g., machinery for crop residue incorporation into soils or no-till seeing equipment.

Crop residue retention with no-tillage is mostly practiced in the North America and about 40% of the cropland across the United States alone is cultivated with no-till practice [92]. This method has many advantages for the soil such as cooling effect, increased moisture, source of carbon, and erosion protection. However, this method also finds some negative implications for example, microbial infestation, formation of phytotoxins and nutrient immobilization. This results in a reduced yield which may warrant additional use of agricultural chemicals [92,93]. For improving the soil organic matter, crop residue is incorporated into the soil by plowing. Adding nitrogen fertilizers while plowing at a depth of 20–30 cm can enrich the soil with humus and prevent nitrogen depression [92].

The National Policy for Management of Crop Residue [7] specifically mentions in-situ management through methods such as direct incorporation into soils and mulching as methods that should be promoted in India not only to control crop residue burning but also to prevent environmental degradation in the croplands. Any specific follow-ups or government-supported interventions since the establishment of this national policy, has not yet been reported in the literature. However, it is worth noting that the National conference on Agriculture for Kharif Campaign that took place in 2017, re-emphasized on the same facts and listed mechanization practices to avoid crop residue burning among the recommendations made by the focus groups [94]. 

## 7. Discussion

In the previous sections of this manuscript we showed that crop residue burning has become an environmental catastrophe, not only for India but for the Asian region as well. Then the policy and implementation steps taken by the Indian government were briefly presented. Using crop residue for making compost/biochar or incorporating into soils were also briefly introduced as they are three key technical solutions, that have not yet been widely considered by the policy sector in India. The sequence of the process of understanding the crop burning issues, looking for potential solutions, and implementation the solutions, seem very logical. However, the smog experienced by millions of people in the country (Figure 1) each year clearly suggests that the crop burning issue has not been sufficiently addressed by any of the previous interventions. The question that needs to be asked is “why not?” The answers to this question will help identify better alternatives for implementation. The key policy-related and/or functionality issues identified based on above analysis are presented in the following sub sections.

### 7.1. The Need for a Running Mechanism

When MSW is generated in households it is properly managed. This is in line with the popular term “Polluter Pays” used in the environmental law which simply explains who should be responsible for producing pollution [95]. Fortunately, community living has already formed a mechanism to manage MSW. The MSW generated by the households in a community is collected, treated and disposed (or at least supposed to be) by the municipality. This implies that there exists a known process to handle the MSW, irrespective of how efficient or sustainable it is. The responsibility of keeping the community clean and safe rests with the municipality and the residents pay taxes and/or other fees as their contribution. It is also worth noting that before intervention by local and municipal governments, MSW was also burned in urban and peri-urban communities.

When crop residue is produced by the farmers, who should take the responsibility of managing it? As per the Polluter Pays principle, it should be the farmer. This often works well with large-scale agricultural businesses, and especially in the developed countries where environmental laws are strictly enforced. However, when it comes to small-scale farming in developing countries, the individual farmers do not have the capacity, or the means to handle their own waste. Going by the MSW example, what is lacking here is a mechanism of an organized effort, such as the municipality, to manage the crop residue. However, government intervention could provide the necessary support for the farmers in establishing an organized network. For example, the local government/municipality can establish a service to manage the crop residue for a reasonable fee charged to the farmer. Until farmers get used to the concept, it is even worth considering providing such service at a government subsidized price.

Based on the needs of the community or the region, the Government agencies can offer different options such as to collect and transport the crop residue from the fields to where it is needed/utilized as a raw material such as a composting, biogas, biochar manufacturing plant. Alternatively, the same entity can establish a service to rent the machinery for those who need equipment to incorporate the crop residue into the soil before the next season. The local government does not necessarily have to take the responsibility. Instead it can be delegated to a community organization such as a farmers’ association, based on the educational and organizational skills of the farmer community. This method has been in use for many years in some water-stressed countries where water distribution must be overseen and controlled. Some of the examples of community involvement in managing recycled wastewater as irrigation water is described in Hettiarachchi and Ardakanian [96].

### 7.2. Empowering Stakeholders

As discussed, before, the Indian government has initiated some pilot projects to raise awareness about crop residue burning and to promote its sustainable utilization as a resource. While these efforts should be praised, one should also question why the efforts have not made any significant impact yet. One reason could be due to the difference between how much work is done by the government versus how much of it was felt or understood by the farming communities. Educating and empowering the farming stakeholders are crucially important steps to make a significant impact. While there is information on implementation of pilot projects, the literature does not provide details on how these projects were communicated to the stakeholders. As of now the thinking of the farmers has been shaped up by what they have seen for generations: they are only responsible for producing crops, but the crop residue is not their responsibility and it is ok to get rid of it with the least cost option. This thinking needs to change, and the farmers should feel responsible for the residue they produce, which is only possible through proper awareness raising campaigns. However, raising the technical knowledge does not mean much until it is packaged with a practical solution to answer their questions on how to handle the crop residue without costing a fortune, or even better, how to make money out of it by using it as a resource. For example, technical knowledge on how to incorporate residue into soils and how much nutrients they can receive by that, will not make a significant impact, when they find out the equipment that they must rent for such operations cost thousands of Rupees (14 USD). The farmers also should be educated about the advantage of reduced agrochemical cost due to the utilization of crop residue in agricultural land. Therefore, awareness raising campaigns should always run parallel to implementation of a practical solution that empowers them not only technically, but also economically.

### 7.3. Avoid Sectorial Thinking: Focus on Nexus Thinking

It is true that the culprit is in the agricultural sector. However, is the issue completely an agricultural issue? Based on what was discussed in the previous paragraphs, crop residue burning is an issue that goes way beyond agriculture. Some of the issues such as the environmental impact is clearly visible, thanks to smog. But for the farmers who cannot afford to spend more money on proper management of crop residue, it has always been an economic issue which is relatively invisible. When the price for equipment rental is thousands of rupees (14 USD) versus the price of a box of matches is a just a few rupees (0.01 USD), from the economic standpoint of the farmer, it is an easy decision to make even if they are knowledgeable about the environmental damage it can cause.

However, what really happens in the big picture is that the farmers are burning a resourceful biomass due to a combination or economic and social issues such as lack of education or awareness. Even though crop residue burning touches upon many sectors, such as environment, agriculture, economy, social aspects, education, and energy, the governmental efforts are mainly revolving only around the agriculture and energy. This sectorial thinking does not help much as exhibited by the slow progress with the previous governmental interventions. Waste material that can be resourceful to the agriculture (soil/food) and the energy sectors gets wasted simply because of sectorial thinking.

This is where the government of India can benefit on the emerging concept of nexus thinking in managing environmental resources. What nexus thinking promotes is a higher-level integration that goes beyond the disciplinary boundaries [97]. One excellent example is the wastewater recycling. Wastewater that originates in the waste sector, is used by many water stressed countries for irrigation purpose after treatment (and in some unfortunate cases, without treatment). This way waste is helping to alleviate the water supply issues faced by the water sector and water demand issues in the agricultural sector. In addition, crops also benefit from the nutrients recycled though wastewater, when used wisely [96]. Compost, biochar, or biogas can be best examples to explain how the nexus thinking can be put to good use while combatting crop residue burning.

## 8. Summary and Conclusions

The mechanization in farming practices, increased share of arable areas for farming with new irrigation schemes, and the use of agrochemicals have contributed to the exponential increase of agricultural production as well as agricultural waste in many countries. The sustainable management of agricultural waste has become a great challenge, especially for developing countries such as India with an increasing population, production rates and economic growth. Crop residues are one branch of agricultural wastes that have posed especial challenges due to their vast volume and lack of capacities to manage them. Taking the fact into account that rice and wheat that usually produce the majority of crop residue being the major staples of India, the large-scale cultivation of these crops to feed the ever-increasing population has obviously led to generation of large quantities of crop residue, that the country is not able to cope up with.

On an average 500 Mt of crop residue is generated yearly in India. While a majority of it is used for fodder, raw material for energy production, etc., still there is a huge surplus of 140 Mt out of which 92 Mt is burnt each year, mainly in the northern states such as Punjab, Haryana and Uttar Pradesh. Especially the small-scale farmers resort to burning of crop waste as it is an inexpensive alternative due to the lack of technical awareness and lack of proper disposal opportunities. Large scale burning of crops increases CO_2_, CO, N_2_O and NO_x_ in the atmosphere and has led to shocking increase in the air pollution. There was an alarming deterioration of the air quality in the northern India to nearly twice the permissible Indian standard and ten times higher than the WHO standard.

The Indian Government has attempted many interventions to curtail the amount of crop residue burning through different campaigns. May such past attempts involved advocacy and encouragement to use crop residue in the energy sector as a raw material. The Indian Agricultural Research institute (IARI), Indian Ministry of New and Renewable Energy (MNRE) are continuously promoting research and innovative measures to handle crop waste without burning. The National policy for management of crop residue (NPMCR) recently formulated by the Central Government, has laid out policies and regulations to be undertaken by the local agencies to curb crop burning and initiatives towards sustainable management practices. As a result, the National Remote Sensing Agency (NRSA) and the Central Pollution Control board (CPCB) now monitor crop burning through aerial surveillance and penalize farmers who burn crops. However, there is little evidence in the publish literature to support the effective control of the situation, most likely due to the lack of education, awareness programs, and stakeholder engagement in the implementation of the policies and initiatives. Continued air pollution especially in the months of November and December suggest that above policies have not fully prevented crop burning.

The real reasons behind the crop residue burning have more socioeconomic roots rather than agricultural or waste management ones. Any solutions involving long-haul transportation, expensive technology, or high capital investment are less likely to succeed. In this context, sustainable solutions that involve methods to feed the nutrients in the crop residue back into the same crop lands have better promise to be successful. Relatively overlooked bio-based products such as biogas, biochar and in-situ management with mechanical intensification are recognized as viable option for crop waste utilization. Large scale harnessing of methane gas from the waste, through biogas plants should be practiced. The Government agencies and private enterprises could develop a natural gas grid to utilize this bio gas. Guidelines could be formulated for composting in rural areas and enforce on all farmers through farmers association. The mechanization in harvesting can considerably reduce crop residue, and the equipment needed could be rented or given in subsidy by the local bodies to the farmers.

There are three key policy-related and/or functionality issues related crop residue management that need to be taken into consideration for any future interventions. They are: (1) The need to think of a self-running mechanism, rather than isolated ones; (2) Empowering stakeholders; and (3) Avoiding sectorial thinking, and if possible, lean towards nexus thinking. Individual small-scale farmers do not have the capacity to establish a long-lasting solution. The local government, the municipality, or a farmers’ association should fill this void and launch community programs to assist such as equipment rentals, waste transportation, and possible linking of waste to where it can be needed as raw materials. Educating the farming community and other related stakeholders is crucially important to bring them out of generational thinking that they are used to that the waste management is not their responsibility. It is even more important to empower them with technical as well as socioeconomic assistance. They should be educated about the advantage of reduced agrochemical cost due to the utilization of compost and the extra revenue they can receive through other type of recovery programs such as energy production. The last, but perhaps the most important piece of the puzzle is the sectorial thinking of the curtailing of the crop residue burning issues only to agricultural sector and energy, even though it touches upon many other sectors, such as environment, economy, social aspects, and education. This sectorial thinking can be overcome by embracing nexus thinking, which promotes a higher-level integration that goes beyond the disciplinary boundaries.

## Figures and Tables

**Table 1:** Agricultural waste generation in India compared to other select nations in the same region [7,8].

Country	Agricultural Waste Generated (million tons/year)
India	500
Bangladesh	72
Indonesia	55
Myanmar	19

**Table 2:** Crop residues produced by major crops [12,13].

Source	Composition
Rice	Husk, bran
Wheat	Bran, straw
Maize	Stover, husk, skins
Millet	Stover
Sugarcane	Sugarcane tops, bagasse, molasses

**Table 3:** Crop Production Estimate of Major Crops in India [7]

Crop	Estimate of Production (Mt)
Rice	105
Wheat	94
Sugarcane	361
Oil seeds	30
Cotton	35
Jute	11
Pulses	17
